# Cross-Talk between Ciliary Epithelium and Trabecular Meshwork Cells In-Vitro: A New Insight into Glaucoma

**DOI:** 10.1371/journal.pone.0112259

**Published:** 2014-11-12

**Authors:** Natalie Lerner, Elie Beit-Yannai

**Affiliations:** Department of Clinical Biochemistry and Pharmacology, Ben-Gurion University of the Negev, Beer-Sheva, Israel; University of Western Australia, Australia

## Abstract

**Purpose:**

It is assumed that the non-pigmented ciliary epithelium plays a role in regulating intraocular pressure via its neuroendocrine activities. To test this hypothesis, we investigated the effect on a human trabecular meshwork (TM) cell line (NTM) of co-culture with a human non-pigmented ciliary epithelium cell line (ODM-2).

**Methods:**

The cellular cross-talk between ODM-2 and NTM cells was studied in a co-culture system in which the two cell types were co-cultured for 5 to 60****min or 2, 4 and 8****h and then removed from the co-culture and analyzed. Analyses of the ERK and p38 mitogen-activated protein kinase (MAPK) pathways and of the activity of TM phosphatases and matrix metalloproteins (MMPs) were performed. Acid and alkaline phosphatase activity was determined by the DiFMUP (6, 8-difluoro-4-methylumbelliferyl phosphate) assay. MMP levels were determined by gelatin zymography.

**Results:**

Exposure of NTM cells to ODM-2 cells led to the activation of the MAPK signal transduction pathways in NTM cells within 5****min of co-culture. Phosphorylation of ERK1/ERK2 and p38 peaked at 10 and 15****min and then decreased over time. Interaction between ODM-2 and NTM cells promoted the expression of MMP-9 in the NTM cells after 4****h of co-culture.

**Conclusions:**

Our findings provide support for the hypothesis that crosstalk does indeed take place between ODM-2 and NTM cells. Future studies should be designed to determine the relationship between the MMP system, MAPK kinases and phosphatases. Manipulation of these signaling molecules and the related NTM signal transduction pathways may provide targets for developing improved treatments for glaucoma.

## Introduction

According to the prediction of a general increase in age-related diseases in the coming years, it is expected that the number of glaucoma patients worldwide will reach 80 million by 2020, despite current advances in therapy [1]. Glaucoma is characterized by the ongoing deterioration of the retinal ganglion layer and worsening of visual field defects, accompanied by changes in the optic nerve head. High intraocular pressure (IOP) has long been considered the most important risk factor for the onset and progression of glaucoma, and therefore pharmacological and surgical treatments have focused on lowering the IOP. However, while IOP management is the only effective glaucoma treatment, it cannot always prevent ongoing nerve damage, and it is still unclear which aspect of IOP (mean, peak, or diurnal) is the factor most relevant to the disease progression. Even normal tension glaucoma patients [2] benefit from IOP lowering-therapy, thus suggesting that relative, rather than absolute, metrics (peak/mean/diurnal) affect the progression of the disease.

Since the FDA approval of prostaglandin analogues in 1996, no new drug families for the treatment of glaucoma have been introduced into clinical practice, despite increasing awareness of the need for new approaches to glaucoma therapy [3]–, with emphasis on neuroprotection. One current avenue of research focuses on the aqueous humor (AH) drainage system [7]. AH produced and released by the ciliary epithelium to the posterior chamber travels through the pupil to the anterior chamber, finally leaving the eye through the trabecular meshwork (TM) tissue to Schlem’s canal or through the uveo-sclera outflow system. The Rho-associated kinase (ROCK) inhibitors that are currently in clinical trials [8] actby relaxing the TM through inhibition of the actin cytoskeleton contractile tone of smooth muscle. This relaxation results in increased aqueous outflow directly through the TM, resulting in a lowering of the IOP [9].

In addition to its role in pressure maintenance, the AH was previously considered to be responsible for nutrient supply and waste product clearance, but it is now realized that the AH is also a rich source of dozens of active protein nucleotide and messenger molecules and vesicles [10]–[15]. However, the exact roles of these components remain, at least partially, obscure. In 2007, Coca-Prados and Escribano hypothesized that the ciliary epithelium acts in a paracrine manner by releasing active factors capable of affecting the TM [16]. In keeping with this hypothesis, we set out to demonstrate the possible AH-mediated influence of the ciliary epithelium on the TM. We suggest that active components released by the non-pigmented ciliary epithelium are transferred by the AH to the TM cells, where they exert their influence.

The active proteins of the AH whose levels and activity are affected by elevated IOP include kinases [17] and phosphatases [11]. The kinases act to phosphorylate either tyrosine or serine/threonine residues, which affects the function of many signal transduction proteins. The regulation of phosphorylated proteins is then directed by three main families of phosphatases–protein-tyrosine phosphatases (PTP), serine/threonine phosphatases (PP) and dual-specificity phosphatases that cleave both PTP and PP. There is a delicate balance between kinase and phosphatase activity, which plays a role in signaling pathway outcome Dephosphorylation of specific phosphorylated sites can inhibit kinase activity. Several human diseases have been attributed to disturbances in Kinase-phosphatases balance, including cancer, diabetes and inflammation [18]. It has been suggested that in the eye increased phosphatase activity in the TM and/or the ciliary muscles could contribute to lowering of the IOP, thus making the TM a possible target for new anti-glaucoma therapies [19].

Among the known targets of oxidant-mediated signaling is the mitogen-activated protein kinase (MAPK) cascade, which comprises highly conserved serine/threonine kinases, connecting cell surface receptors to regulatory targets in response to various stimuli [20]. The MAPK pathway can be stimulated by a number of factors contributing to the pathophysiology of primary open-angle glaucoma, including different types of mechanical [21]–[23] and oxidative [24] stress. In addition, there is accumulating evidence that members of the MAPK family play a critical role in TM cell apoptosis [25]. Among the MAPK proteins, p38 has been shown to mediate TGF-β2 up-regulation via the gene *SPARC*, one of the most highly transcribed and ubiquitous genes in the TM, especially in the juxtacanalicular region–the region believed to be the anatomic location of outflow regulation and the part of the AH where outflow resistance is maximal [26].

Matrix metalloproteinase (MMP) has been shown to be one of the most important activated proteins downstream of the MAPK activation and its activity is regulated by the kinase-phosphatases balance. The promoter regions of inducible MMP genes are regulated by MAPKs. This MAPK regulation depends on the nature of the extracellular stimuli, namely, the presence of different growth factors, cytokines, and IFγ [27], [28]. MMPs play a powerful role in the resistance of the drainage system to AH outflow [21] and, as such, have also been suggested as a potential intervention site for glaucoma therapy [29]. A number of studies have provided evidence for the direct influence of MAPK signaling on the activity of MMPs within the TM [13], [30]. In the present study, we examined in****vitro the presence of soluble factors involved in cross talk of between non-pigmented ciliary epithelium and TM cells with the aim to shed light on the MAPK-phosphatases balance and its consequences for the activity of the MMPs.

## Materials and Methods

### Cell lines

A human normal trabecular meshwork (NTM) cell line [31] was generously donated by Alcon Laboratories, Texas, USA. A human non-pigmented ciliary epithelial cell line (ODM-2) [32], [33] and a ciliary muscle cell line (HCM) [34] were kindly supplied by Prof. Miguel Coca Prados, Yale University, MA, USA. All cell lines were cultured at 37°C and 5% CO_2_ in Dulbecco’s modified Eagle’s medium (DMEM) supplemented with fetal bovine serum, penicillin, streptomycin, and glutamate, as previously described [31], [35].

### Establishment of co-cultures

**Figure 1:**
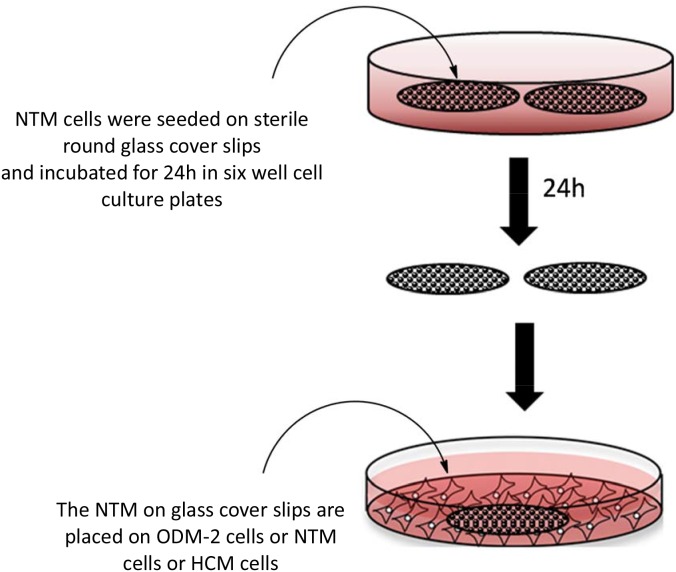
Schematic description of the co-culture model. Sterile round glass cover slips were placed in six-well plates, and seeded with NTM cell. The cultures were incubated for 24****h to allow the NTM cells to attach to the cover slips. In parallel, ODM-2 as the tested cells, HCM and NTM cells as controls were seeded in 60-mm cell culture dishes. After 24****h, two cover slips with their adhered NTM cells were introduced into each cell type culture dish.

Sterile round glass cover slips (Marienfeld, 22****mm) were placed in six-well plates, and 3****ml of NTM cell suspension was pipetted onto each coverslip (1 million cells/well; ∼100,000 cells/cm^2^). The cultures were incubated in DMEM at 37°C in 5% CO_2_/95% air for 24****h to allow the NTM cells to attach to the cover slips. In parallel, HCM, ODM-2, and NTM cells were seeded in 60-mm cell culture dishes (2.5 million cells in 5****ml per dish; ∼90,000 cells/cm^2^). After 24****h, two cover slips with their adhered NTM cells, but free of conditioning medium, were introduced into each cell culture dish with the cell-coated side of the coverslip facing upwards (Figure 1). Since the study focused on cell-cell communication, it was not necessary to determine the basal levels of the TM cells or the base-line activity of the tested proteins. As a control for the possible ODM-2:NTM signaling effects, we used the following co-cultures: ODM-2:HCM to verify the specificity of the signaling, and NTM:NTM to exclude contact effects. All the co-cultures were incubated for various periods of time, as indicated below. At the end of the incubation, the coverslips with the attached cells were removed, and analyzed as described below. The experiments were performed in duplicate and repeated independently three times.

### Gelatin zymography and western blot analysis

Following co-incubation with ODM-2 cells, the NTM cells were lysed with buffer containing 20****mM Hepes (pH****7.4), 150 mM NaCl, 1****mM EGTA, 1****mM EDTA, 10% glycerol, 1 mM MgCl_2_, 25****mM NaF, 1****mM Na_3_VO_4_, and protease inhibitors. After incubation on ice for 30****min, the lysates were sonicated for 10 centrifuged at 12,000****g at 4°C, and the supernatant was collected. The supernatant was analyzed for protein concentration by using the Bradford method [36]. For gelatin zymography, 20****µg of total protein from the lysates were electrophoresed through a 10% SDS-PAGE gel polymerized with 0.1% gelatin. Gels were washed twice, each time for 1****h in 2.5% Triton X-100, to remove SDS. Gels were then incubated for 16****h at 37°C in a developing buffer containing 50****mM Tris, 200****mM NaCl, 5****mM CaCl_2_, and 0.02% Brij-35. Gels were stained with 30% methanol, 10% acetic acid, and 0.5% w/v Coomassie Brilliant Blue for 30****min, followed by distaining and then, dried between sheets of cellophane (Gel drying frames, Sigma). Gelatinolytic activity was manifested as horizontal white bands on a blue background. The proteolytic digestion of gelatin was quantified using an EZQuant-Gel 2.1, EZQuant Biology Solution. Three independent experiments were performed in duplicate.

For Western blot analysis, 20****µg of protein extracted from NTM cells were mixed with Laemmli buffer (Bio-Rad) containing 0.1% β-mercaptoethanol, boiled for 5****min, and separated on a10% SDS-PAGE gel. Proteins were transferred to nitrocellulose membranes and probed with primary antibodies against phospho-specific ERK1/2 (Sigma M9692, mouse monoclonal; 1:3,000 dilution), total ERK1/2 (Sigma M5670, rabbit polyclonal; 1:20,000 dilution), anti-phospho-p38 (Sigma M8177, mouse monoclonal; 1:2,000 dilution), and total p38 (Sigma M0800, rabbit polyclonal; 1:10,000 dilution). β-actin was determined using anti β-actin (Sigma A2228, mouse monoclonal 1:4000 dilution). The membranes were then incubated with secondary antibodies (anti-rabbit GE Health life sciences Na934v or anti-mouse Jackson 112035062) for 1****h at room temperature, and the immunocomplexes were detected with an enhanced chemiluminescence reagent (Amersham Pharmacia Biotech, Buckinghamshire, England) for 1****min, followed by exposure to Kodak X-ray film (Rochester, NY). The films were scanned, and densitometry was analyzed by EZQuant-Gel software (EZQuant Biology Solution). The results from replicate experiments, expressed as percentages of the control, were pooled and averaged. Three independent experiments were performed in triplicate.

### Phosphatase assays

Fluorogenic substrates based on 4-methylumbelliferone (4-MU) have been widely used for the detection of glycosidase and phosphatase activities, especially alkaline phosphatases. 6, 8-Difluoro-4-methylumbelliferyl phosphate (DiFMUP) is a fluori­nated derivative of 4-MU with a lower pKa value, which makes it suitable for the detection of both alkaline and acid phosphatases. Hydrolysis of DiFMUP by a phosphatase results in the release of fluorescent DiFMU, which can be easily followed in continuous mode by a fluorescence reader.

NTM cells adheed to coverslips were co-incubated with ODM-2 cells for 5, 10, 15, 30, or 60****min. The NTM cell cultures on the coverslips were then removed from the co-culture and washed in PBS. Cells were lysed in a buffer containing 20****mM Tris (pH****7.5), 6****mM MgCl_2_, 100****mM NaCl, 0.1****mM EGTA, 1****m EDTA, and 0.5****mM dithiothreitol. Lysates were centrifuged, and the supernatants were retained. The assay was carried out in black flat-bottomed 96-well plates in a total reaction volume of 100****µl. The total 100****µl of reaction mixture included 5****µl of sample, 25****µl of DiFMUP (Biorad) 200****µM diluted in DMSO, 70****µl of acidic buffer (sodium acetate 50****mM, EDTA 1****mM, DTT 21****mM, NaCl 100****mM; doubly distilled water to 10****mL, pH****5.5) or alkaline buffer (Hepes 50****mM, EDTA 1****mM, DTT 2****mM, NaCl 150****mm, doubly distilled water to 50****mL; pH****6.9). Blanks included wells without samples and wells without DiFMUP. The supernatants were incubated with 10****µM of DiFMUP, for 10****min at 37°C with intermittent mixing in a fluorescence plate reader, infinite microplate reader (M200; Tecan Group Ltd.) and the data capture software (Bio-Lynx) with 360****nm excitation and 460****nm emission filters. Three independent experiments were performed in triplicate.

### Statistics

All the data acquired in the study was compared to that for NTM:NTM cells at the shorter incubation time tested (5****min or 4****h) as the reference point. Analysis of data was performed using a one-way ANOVA (InState 3.0, Graphpad Software).

## Results

### MAPK

**Figure 2:**
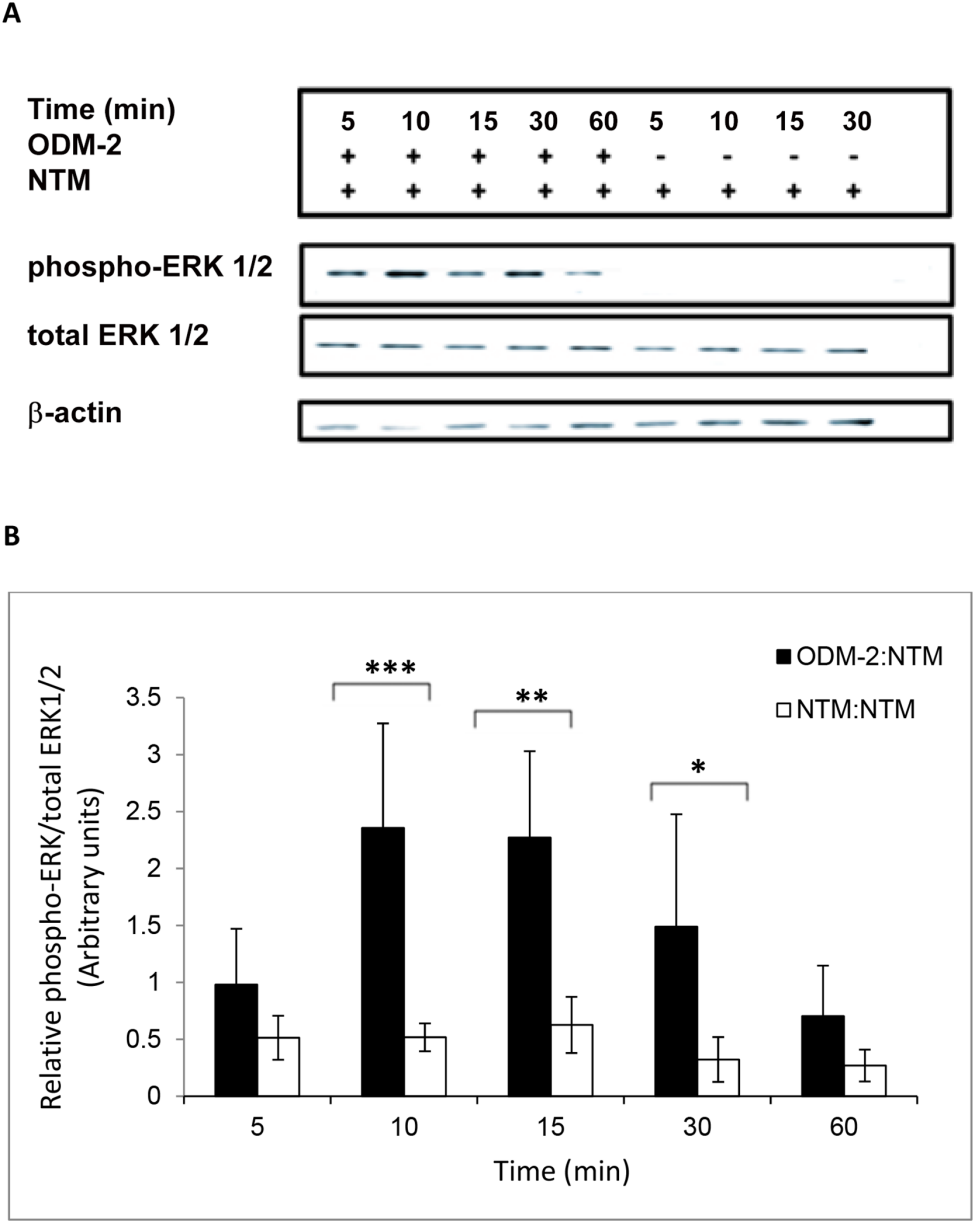
ERK1/2 activation in NTM cells co-cultured with ODM-2 cells for 5, 10, 15, 30, 60 min. Total protein was extracted from NTM cells. Phosphorylated and total ERK1/2 proteins were analyzed by Western blot. (A) Representative western blot film. (B) A significant increases in ERK activation in NTM cells following co-culture with ODM-2 cells. The bar graph represents the means ± SD of three independent experiments performed in duplicates. One-way ANOVA (***p<0.001, **p<0.01,*p<0.05).

To evaluate the cross-talk between the ciliary epithelium and the TM, the two cell lines were co-cultured in Petri dishes. A co-culture of NTM cells grown on cover slips introduced to NTM cells was used as control. After removal of the NTM cells on the cover slips at different time points following initiation of the co-culture, NTM proteins were analyzed for total Erk (tErk) and its active phosphorylated form (pErk). The ratio between the two represents the activity of the Erk branched pathway of the MAPK. A significant increase (ANOVA p<0.001) in Erk activation vis-à-vis the control treatment was found from 10****min following co-culture up to 30****min (Figure 2). This increase in Erk activation peaked at 10****min, demonstrating a transient activation with a bell shape. The phosphorylated Erk/total Erk ratio did not change with time in HCM cells exposed to ODM-2 cells (Figure S1).

**Figure 3:**
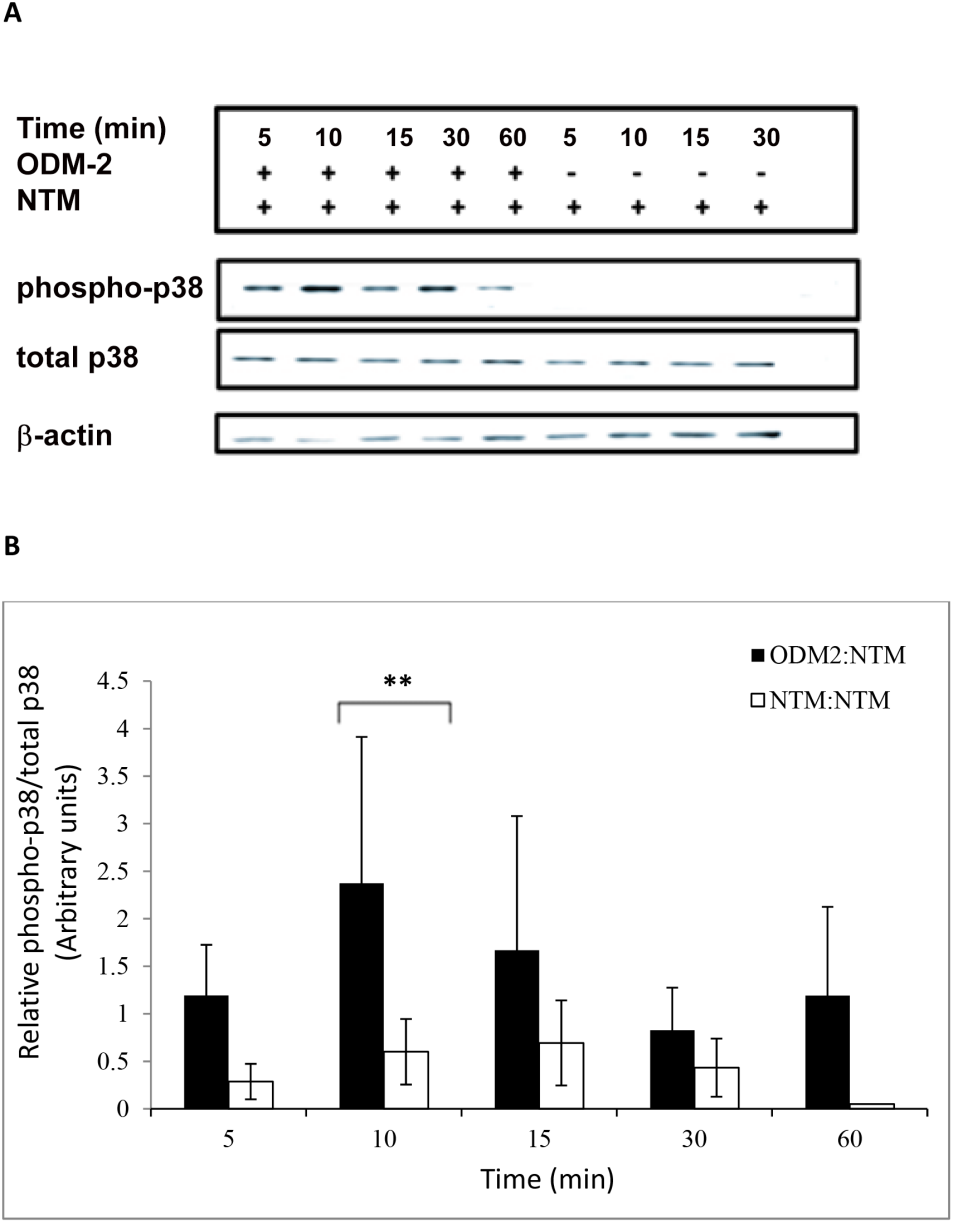
p38 activation in NTM cells co-cultured with ODM-2 cells for 5, 10, 15, 30, 60 min. Total protein was extracted from NTM cells. Phosphorylated and total p38 proteins were analyzed by Western blot. (A) Representative western blot film. (B) A significant increases in p38 activation in NTM cells following co-culture with ODM-2 cells. The bar graph represents the means ± SD of three independent experiments performed in duplicates. One-way ANOVA (**p<0.01).

To evaluate the communication between the ciliary epithelium and the TM, the two cell lines were co-cultured in petri dishes. A co-culture of NTM cells with NTM cells was used as control. The degree of activation of the p38 pathway of the MAPK was significantly elevated (ANOVA p<0.01 for 10****min) in the ODM-2:NTM co-culture vis-à-vis the NTM:NTM control (Figure 3). A trend of elevated activation can be seen from 5****min of co-culture through 60****min. The ratio of phosphorylated p38/total p38 did not change with time in HCM cells exposed to ODM-2 cells (Figure S2).

### Acid and alkaline phosphatase activity following co-culture of NTM cells with ODM-2 cells

**Figure 4:**
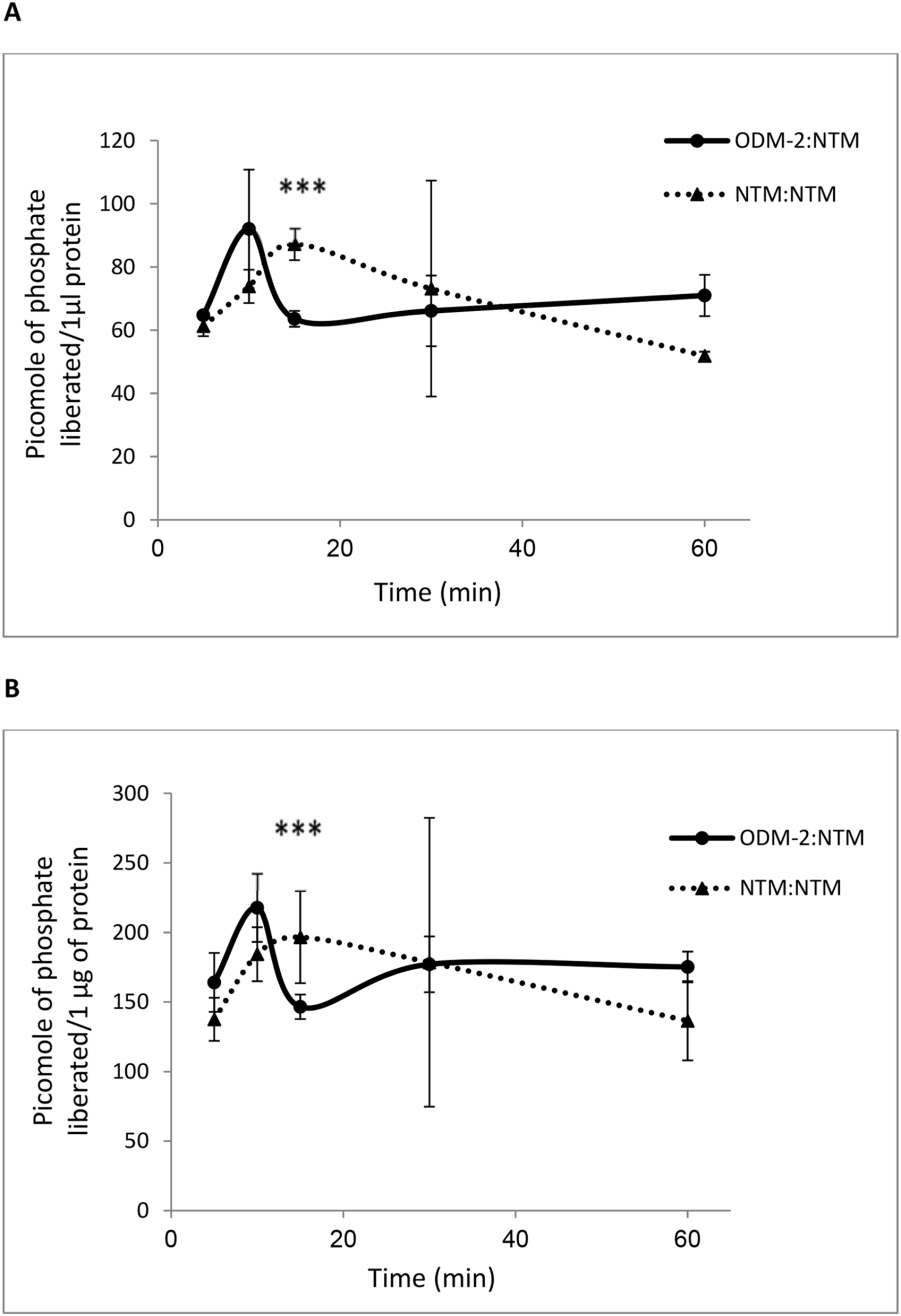
Alkaline and acid phosphatase activity of NTM cells following co-culture with ODM-2 cells for 5, 10, 15, 30 or 60 min. NTM:NTM co-culuure was used as controls. (A) Alkaline phosphatase and (B) acid phosphatase activity determined by the DiFMUP assay. The quantity of DiFMU is expressed as phosphate liberated, since equivalent amounts of DiFMU and phosphate are generated in the reaction. Means of two independent experiments performed in triplicate; one-way ANOVA (***p<0.001).

We determined the activity of alkaline and acid phosphatases as indicative of serine/threonine phosphatases and tyrosine phosphatases, respectively, by the fluorescent DIMFUP method. The activity of the two families of phosphatases peaked at 10****min and then dropped markedly at 15****min (ANOVA p<0.001) (Figure 4). At 30****min post co-culture, the activity of the NTM cells exposed to ODM-2 cells was similar to that of the control, namely, NTM cells co-cultured with NTM cells. While the phosphatase activities of the ODM-2:NTM co-cultures remained steady between 30 and 60****min, a continuous non-significant decline in phosphatase activity was found in the control group.

### Activity of MMPs following co-culture of NTM cells with ODM-2

**Figure 5:**
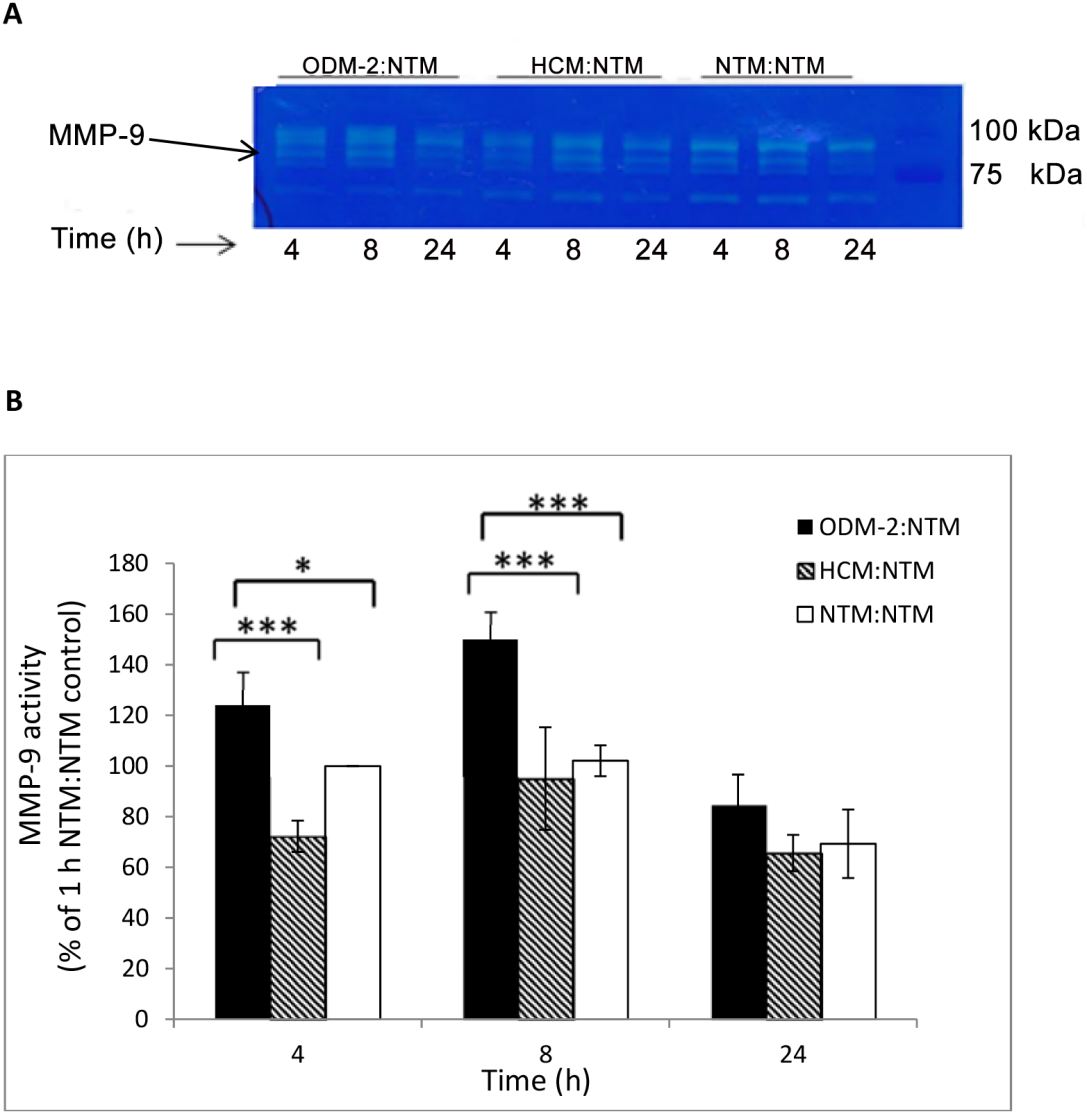
MMP-9 activity following ODM-2 and NTM cells co-culture. Gelatinolytic activity of MMP-9 (92****kDa) in NTM cells was examined by gelatin zymography after 4, 8, and 24****h of co-culture with ODM-2 cells. As controls, NTM cells were co-cultured with HCM or NTM cells. (A) Zymogram of co-culture groups. (B) MMP-9 quantitative analysis of NTM cells co-cultured with ODM, NTM or HCM cells for 4, 8 and 24****h. The bar graph represents the means ± SD of three independent experiments performed in duplicate; one-way ANOVA (*p<0.05, ***p<0.001).

MAPK activation is known to regulate the expression of different MMPs, which contribute to the AH outflow by decreasing the resistance of the extracellular matrix to AH drainage. To verify the specific response of NTM cells to co-culture with ODM-2 cells, two controls were used in these experiments, the NTM:NTM co-culture to control for contact effects and the HCM:NTM co-culture to control for the specificity of the signaling effects. The method facilitates the detection of the non-active form of the enzymes (pro-enzymes) and the post-cleavage active enzymes with gelatinolytic activity. The NTM cell lysate obtained after the co-culture contained MMP-9 and MMP-2. These two MMPs, which have previously been reported in TM cells, were identified according to their molecular weights and migration distance on the zymography gel. MMP-2 activity was barely measurable, but MMP-9 activity was easily quantified (Figure 5). A significant increase (ANOVA p<0.001) in MMP-9 activity following NTM exposure to ODM-2 cells was found at 8 h post co-culture. Although the activity of MMP-9 was higher for the HCM:NTM control co-culture than for the NTM:NTM control co-culture, the difference was not significant and the activity in both controls was lower than that in the ODM-2:NTM co-culture. MMP-9 activity in the NTM cells following ODM-2:NTM co-culture exhibited a bell-shaped curve with a peak at 8****h.

## Discussion

Elevated IOP, known to be the main risk factor for the development of primary open-angle glaucoma, can be controlled by medication targeting the production and/or drainage of the AH. The role of MMPs within the drainage system was demonstrated in the study of Bradley et al. [37]. These authors concluded that the balance of MMPs and their tissue inhibitors, TIMMPs, that controls the endogenous extracellular matrix turnover, is responsible for the maintenance of TM outflow resistance. However at present, there areno drugs that target the balance of MMPs and TIMMPs within the TM, the main AH drainage pathway (although some currently available prostaglandin analogues that are effective IOP lowering agents exert their pharmacological action by targeting the MMPs involved mainly in the uveo-scleral AH drainage).

In the present study, the gelatinolytic activity of MMPs following TM signaling activation by ciliary epithelium cells was evaluated. We focused on MAPK and phosphatase pathways, although other pathways such as Wnt [38] or Akt [39] might be involved. We report here for the first time that, in a co-culture model of ODM-2 and NTM cell lines, cross-talk between ODM-2 cells and NTM cells appears to promote MMP-9 activity. To date, the mechanism governing the up-regulation of MMPs as the result of interaction between ODM-2 and NTM cells has been hypothesized but not demonstrated [16]. We therefore focused on the elucidation of the signaling pathways that are modulated by the co-culture. Bearing in mind that pigmented epithelium cells, synchronized in****vivo with non-pigmented epithelium cells, in the production of AH. This synchronization might make a significant contribution to the signaling release of non-pigmented ciliary epithelial cells. Our study focused solely on effects of the latter. However, possible contribution of pigmented epithelial cells in the present model will require further research. Although this, study was performed in quite well characterized cell lines [31]–[34], the relevance and the importance of the present findings would be strengthened by their replication in a primary culture.

It is known that the promoter regions of inducible MMP genes are regulated by MAPKs in a manner that depends on the nature of the extracellular stimuli [40]. Our results revealed that interaction of ODM-2 with NTM cells leads to increased phosphorylation of the ERK1/2 pathway and to increased p38 signaling in NTM cells. Taken together, these results suggest that cross-talk between ODM-2 and NTM cells results in signal activation, signifying, in turn, that these pathways may be intimately involved in the regulation of levels of MMPs in the co-culture of NTMs and ODM-2 cells. Recent reports suggest that targeting of MAPKs, in particular ERK1/2 and JNK, may be a potent approach to reduce MMP expression in a variety of cells [41]. Downstream MAPK signaling pathway active molecules, such as c-Jun and C-Fos, translocate to the nucleus to promote transcription directly or indirectly. Indeed, in our preliminary, unpublished data in our laboratory indicated that, c-Jun expression in NTM cells increased in response to the stimuli induced by ODM-2:NTM co-culture. We still do not have a clear picture of the putative soluble transcription regulators of the ERK1/2 and p38 signaling pathways in the ODM-2:NTM co-culture vis-à-vis the NTM:NTM co-culture. The MAPK-MMP pathways appear to be regulated transcrip­tionally by classic mediators, such TNF-α [42], VGEF [43], TGF-β [44] and interleukins [42], all of which, in turn, activate a pleotropic cascade of signaling pathways.

A number of studies have demonstrated the role of neuropeptides in mediating the levels of MMPs [45], [46]. Transcripts (mRNA) for neuropeptides have been found in both dissected ciliary processes and cultured ciliary epithelial cells by Northern blot analysis and RT-PCR. The secreted products of ciliary epithelial cells either in the AH or in the culture medium of cultured ciliary epithelial cells have been detected by radioimmunoassay, suggesting that communication occurs between these two types of tissue. Furthermore, it has been found that neuroendocrine processing enzymes are co-expressed with the neuroendocrine peptides, suggesting that the neuropeptides synthesized in the ciliary epithelium undergo endoproteolytic processing and storage before they are secreted [16]. Coca-Prados [16] proposed that the neuropeptides released by the ciliary epithelium in the AH can serve as messengers to communicate with surrounding tissues in the anterior segment of the eye, particularly, in the TM. It is therefore possible that one or more of the factors described above or some unknown neuropeptide are induced in the ODM-2:NTM co-culture, thereby giving rise to a positive feedback to the ERK1/2 and p38 pathways, which leads to the MMP-mediated degenerative changes in the NTM cells.

While the role of kinases in the regulation of MMPs has been extensively studied, the role of phosphatases in the homeostasis of MMPs remains obscure. Nonetheless, it is now apparent that they are equally important in mediating cell signaling, apoptosis, growth, migration and adhesion. The early report of Holladay et al. that okadaic acid, a protein phosphatase 1 and 2A inhibitor, increases MMP-3 (stromelysin-1) expression in murine keratinocytes [47], [48] has been confirmed and extended by other researchers [49]. The specific mechanisms that contribute to the increase in MMP abundance following phosphatase inhibition remain largely unexplored. It is possible that the increased MMP presence following phosphatase inhibition may simply reflect maintenance of the phosphorylation and hence the activation state of signaling pathways upstream of the transcriptional regulation of the MMPs. For instance, PP2A mediates the dephosphorylation of MAPK kinase 1 and the ERK family kinases [50], which are associated with MMP and TIMMP regulation. More specifically, Westermarck’s group found that okadaic acid-induced expression of MMP-3 is mediated through transactivation of AP-1 complexes containing c-Jun and JunB in HT-1080 cells [47]. However, the process may be more complex than previously thought, as the recent work of Rietz et al. shows that okadaic acid increases the abundance of MMP-9 in fibroblasts through p38-MAPK and is inhibited by isoprenaline via a pathway involving β-arrestin 2, PP2A and an NF-κB binding motif [49].

In the current study, it was observed that within 10–15 min of co-culture with ODM-2 cells, NTM cells revealed increase levels of MAPKs, whereas a complex activity/time response exist in the alkaline and acid phosphatases activity as compared to control. In ODM-2:NTM and the control cells a quick and short up-regulation of general phosphatase activity was found, followed by down-regulation. The changes were more marked in the ODM:NTM co-culture, with different kinetics relative to the control NTM:NTM cells, indicating that processes occurring beyond simple contact stress do exist. The involvement of phosphatases in the ocular drainage system was demonstrated partially in our previous research in AH [11]. The phosphatases response detected is a general analysis reflecting probably several phosphatases activity, and further research clarifying the specific phosphatases involved is required. The changes in phosphatases kinetics may support the crosstalk between MAPK kinases, MMPs and phosphatases. However, more detailed experiments are needed designed to determine the relationship between the MMP system, MAPK kinases and phosphatases, with focus on PP2A phosphatase.

## Supporting Information

Figure S1 **Effect on ERK1/2 activation in NTM cells of interaction between NTM and HCM cells co-cultured for 5, 10, 15, 30, or 60 min.** Following co-culture of the cells, total protein was extracted from NTM cells. Changes in ERK activation in normal TM cells following co-culture with HCM cells. Phosphorylated ERK1/2 proteins were analyzed by Western blot. The bar graph represents the means ± SD of two independent experiments performed in triplicate. One-way ANOVA (NS p>0.05). Phosphorylated Erk/total Erk ratio did not change with time in HCM cells exposed to ODM-2 cells.
(TIF)

Figure S2 **Effect on p38 activation in NTM cells of interaction between NTM and HCM cells co-cultured for 5, 10, 15, 30, or 60 min.** Following co-culture of the cells, total protein was then extracted from NTM cells. Changes in p38 activation in normal TM cells following co-culture with HCM cells. Phosphorylated p38 proteins were analyzed by Western blot. The bar graph represents the means ± SD of three independent experiments performed in duplicate. One-way ANOVA (NS p>0.05). Phosphorylated p38/total p38 ratio did not change with time in HCM cells exposed to ODM-2 cells.
(TIF)
