# Supplementary figures and images for: Cross-Talk between Ciliary Epithelium and Trabecular Meshwork Cells In-Vitro: A New Insight into Glaucoma
Source: PLoS One. 2014 Nov 12;9(11):e112259. doi: 10.1371/journal.pone.0112259 (PMC4229184; doi:10.1371/journal.pone.0112259)

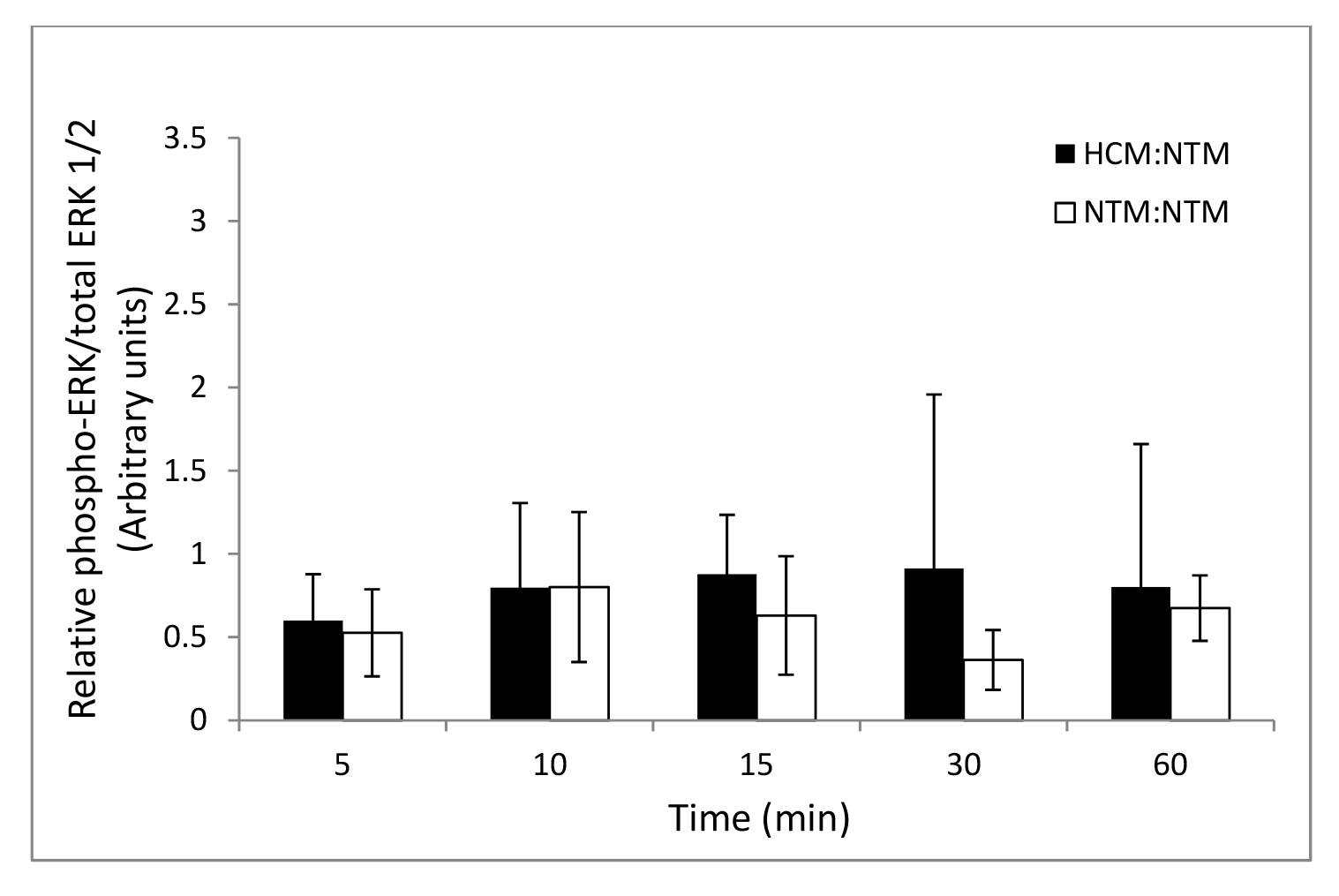

Supplement: Figure S1 — Effect on ERK1/2 activation in NTM cells of interaction between NTM and HCM cells co-cultured for 5, 10, 15, 30, or 60 min. Following co-culture of the cells, total protein was extracted from NTM cells. Changes in ERK activation in normal TM cells following co-culture with HCM cells. Phosphorylated ERK1/2 proteins were analyzed by Western blot. The bar graph represents the means ± SD of two independent experiments performed in triplicate. One-way ANOVA (NS p>0.05). Phosphorylated Erk/total Erk ratio did not change with time in HCM cells exposed to ODM-2 cells. (TIF) [file pone.0112259.s001.tif]

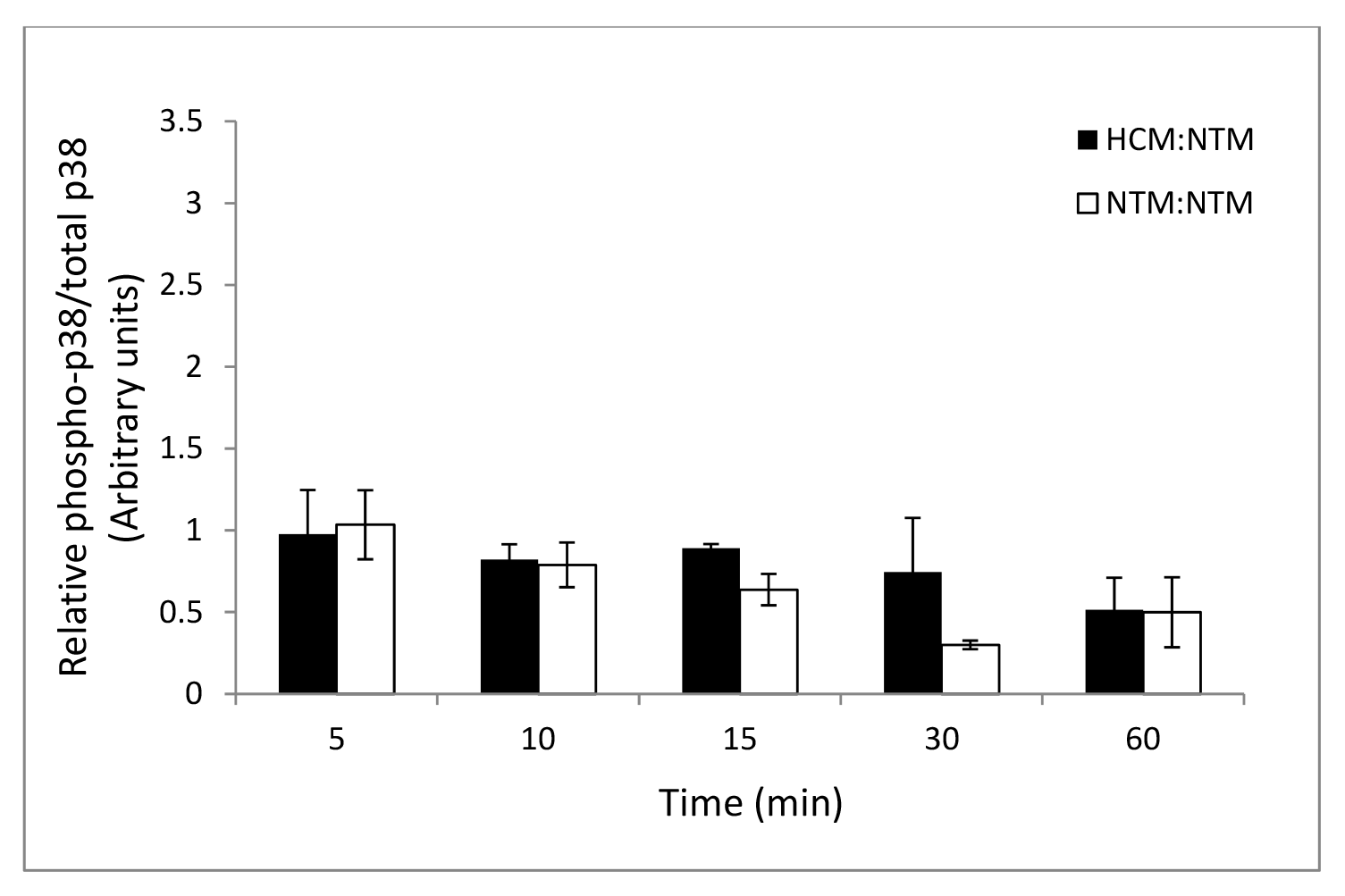

Supplement: Figure S2 — Effect on p38 activation in NTM cells of interaction between NTM and HCM cells co-cultured for 5, 10, 15, 30, or 60 min. Following co-culture of the cells, total protein was then extracted from NTM cells. Changes in p38 activation in normal TM cells following co-culture with HCM cells. Phosphorylated p38 proteins were analyzed by Western blot. The bar graph represents the means ± SD of three independent experiments performed in duplicate. One-way ANOVA (NS p>0.05). Phosphorylated p38/total p38 ratio did not change with time in HCM cells exposed to ODM-2 cells. (TIF) [file pone.0112259.s002.tif]
